# A Short Review on the Impact of Artificial Intelligence in Diagnosis Diseases: Role of Radiomics In Neuro-Oncology

**DOI:** 10.31661/gmj.v12i.3158

**Published:** 2023-12-16

**Authors:** Mohammad Rahimi, Parsa Rahimi

**Affiliations:** ^1^ Student Research Committee, School of Medicine, Mazandaran University of Medical Sciences, Mazandaran, Iran; ^2^ Student Research Committee, School of Medicine, Tehran University of Medical Sciences, Tehran, Iran

**Keywords:** Artificial Intelligence, Early Diagnosis, Neouro-Oncology, Machine Learning, Radiomics

## Abstract

Artificial Intelligence (AI) is rapidly transforming various aspects of healthcare, including the field of diagnostics and treatment of diseases. This review article aimed to provide an in-depth analysis of the impact of AI, especially, radiomics in the diagnosis of neuro-oncology diseases. Indeed, it is a multidimensional task that requires the integration of clinical assessment, neuroimaging techniques, and emerging technologies like AI and radiomics. The advancements in these fields have the potential to revolutionize the accuracy, efficiency, and personalized approach to diagnosing neuro-oncology diseases, leading to improved patient outcomes and enhanced overall neurologic care. However, AI has some limitations, and ethical challenges should be addressed via future research.

## Introduction

Neuro-oncology disease diagnosis is a critical aspect of patient care and management in the field of neurology [1]. With the increasing prevalence of brain tumors and other neurologic malignancies, accurate and timely diagnosis is essential for appropriate treatment planning and improving patient outcomes [2]. Diagnosing neuro-oncology diseases could be challenging due to the intricate nature of the central nervous system and the diverse types of tumors [3]. Also, clinical presentations alone may not provide sufficient information for an accurate diagnosis, necessitating the integration of various diagnostic modalities [4]. Neuroimaging techniques play a fundamental role in the diagnosis of neuro-oncology diseases. Imaging modalities such as magnetic resonance imaging (MRI), computed tomography (CT), and positron emission tomography (PET) allow for the visualization and characterization of brain tumors [5]. These imaging techniques provide valuable information about tumor location, size, morphology, and involvement of surrounding structures, aiding in differential diagnosis and treatment planning [6].

Artificial intelligence (AI) has emerged as a novel technology with numerous applications in various fields [7]. Among them, the diagnosis of diseases is one of the most promising areas where AI has shown immense potential [8]. Also, the use of AI could enhance the approach and address diseases, significantly impacting patient outcomes and healthcare efficiency [9][10].

Radiomics involves the extraction of quantitative features from medical images, such as texture, shape, and intensity, and their analysis using advanced computational techniques [11]. Indeed, radiomics allows for a comprehensive characterization of tumors beyond visual assessment, providing additional information about tumor heterogeneity, aggressiveness, and prognostic indicators [12].

The integration of AI and radiomics holds great promise in revolutionizing neuro-oncology diagnosis. Hence, in this review, the impacts of AI-based diagnosis methods, especially, radiomics in neuro-oncology fields are provided.

## AI Technology and Radiomics Transformed Diagnosis of Neuro-Oncology Diseases

AI and radiomics are transforming the field of neuro-oncology diagnosis, offering new avenues for improved understanding, accurate detection, and personalized treatment of neuro-oncology disorders [13].

One significant contribution of AI in neuro-oncology diagnosis is its ability to analyze neuroimaging data with enhanced speed and accuracy [14]. Actually, AI algorithms are able to process large volumes of medical images, such as MRI scans, CT scans, and PET scans, to identify subtle abnormalities and patterns that may be missed by human observers [15]. Therefore, these algorithms can aid in the early detection and classification of brain tumors, providing clinicians with invaluable insights into tumor characteristics and behavior [16].

Radiomics, on the other hand, focuses on the extraction and analysis of quantitative features from medical images. Indeed, lesion characteristics can serve as valuable imaging biomarkers that help predict tumor aggressiveness, treatment response, and patient prognosis [17]. Hence, the integration of radiomics in neuro-oncology diagnosis enables a more comprehensive and precise understanding of tumor biology, guiding clinicians in making informed treatment decisions.

Also, AI and radiomics contribute to the development of personalized medicine in neuro-oncology [18]. Indeed, by analyzing a patient’s specific tumor characteristics, AI algorithms can assist in tailoring treatment approaches. For instance, they can help determine optimal surgical resection margins, identify the most suitable candidates for targeted therapies or immunotherapies, and predict treatment response or potential side effects [19]. This personalized approach maximizes the chances of successful treatment outcomes while minimizing unnecessary interventions and adverse effects [20].

Furthermore, AI and radiomics enhance collaboration and knowledge-sharing in neuro-oncology diagnosis [21]. By integrating and analyzing large amounts of neuroimaging and clinical data from various sources, AI algorithms can identify similarities and patterns across patients. These insights contribute to the development of robust diagnostic models and decision support systems, enabling healthcare professionals to benefit from collective intelligence and evidence-based practices [22]. The sharing and pooling of data also facilitate ongoing research and the discovery of novel biomarkers and therapeutic targets [23].

Currently, many studies are being conducted in the field of using AI in image analysis to determine the nature of brain lesions. For example, Artzi et al. [24] revealed that by using radiomics analysis based on conventional post-contrast T1-weighted MRI, diffraction between glioblastoma from the metastatic lesion was provided with an accuracy of 85%.

Generally, primary central nervous system lymphoma (PCNSL) is treated with chemotherapy and whole-brain radiotherapy, whereas patients with glioblastomas commonly undergo gross surgical resection followed by chemo-radiotherapy [25]. Hence, differentiating these lesions is an essential step in the treatment approach. Regarding Nguyen et al. study [26], machine learning could be able to distinguish between glioblastomas and PCNSL on radiological imaging by high success rate.

Also, recent studies [27][28] evaluated the role of radiomics in determining the relationship between imaging phenotypes and gene expression patterns (e.g., isocitrate dehydrogenase mutation status), which might allow improved diagnosis, decision-making, and predicting patient outcomes. However, the full potential of AI in this field is not fully known, and as a result, more studies are needed.

## Ethical Concerns in AI-Based Disease Diagnosis

The advancement and integration of AI and radiomics in neuro-oncology diagnosis bring about important ethical considerations that must be addressed for their responsible implementation.

One major concern is the potential bias embedded in AI algorithms and radiomics models [29]. These technologies basically rely on training data, which may be influenced by human biases and demographic imbalances [30]. If the data used for training is not representative of the different populations, the AI models may exhibit biased outcomes, leading to disparities in diagnosis and treatment recommendations. It is crucial to ensure the use of more different and inclusive datasets to mitigate bias and promote equitable healthcare outcomes for all patients [31].

Data privacy and patient consent are also significant ethical concerns in the context of AI and radiomics. The application of large volumes of patient data for training and testing algorithms raises questions about data protection, confidentiality, and informed consent [32]. Strict measures must be in place to ensure the anonymization and secure storage of patient data, as well as obtaining explicit consent for its usage [33]. Transparency in data collection, use, and sharing practices is essential to maintain patient trust and uphold their privacy rights [34].

Another ethical consideration lies in the implementation of AI and radiomics as decision-support tools rather than replacing human expertise [35]. While these technologies offer marked insights and assist in diagnosis and treatment planning, they should be seen as aids to clinicians rather than making autonomous decisions [36]. The final responsibility and accountability still lie with healthcare professionals. It is imperative to provide a balance between the benefits of AI-driven assistance and the expertise of healthcare providers to avoid overreliance or potential abdication of human judgment [37].

## Challenges and Limitations of AI and Radiomics

While AI and radiomics have shown promise in revolutionizing neuro-oncology diagnosis, they also face several challenges and limitations that need to be addressed for their effective implementation.

One significant challenge is the dependence on high-quality and various datasets for training AI algorithms and radiomics models [38]. Obtaining such datasets is often challenging due to privacy concerns, limited availability of annotated data, and the rarity of certain neuro-oncology disorders [39][40]. The lack of different and representative data can limit the development of robust and accurate algorithms, potentially leading to suboptimal diagnostic performance [41]. Efforts should be made to establish comprehensive and curated datasets that encompass various neuro-oncological conditions to improve the generalizability and reliability of AI and radiomics approach [42].

Additionally, the lack of standardized protocols and coordination between different imaging modalities could create a limitation in the field of neuro-oncology diagnosis [43]. Although radiomics relies on extracting quantitative features from medical images, the methods for feature extraction and selection may vary across different institutions and studies. Hence, this inconsistency in protocols can lead to variations in results and impair the reproducibility and comparability of findings [44]. Standardization efforts and the development of quality control measures are necessary to ensure consistent and reliable radiomics analysis in neuro-oncology.

Furthermore, the integration of AI and radiomics into clinical workflows may create practical challenges. Indeed, combining these technologies into routine clinical practice requires extensive validation, seamless integration with existing infrastructure, and wobnbnjrkflow modifications [45]. The adoption of new technologies in healthcare settings demands careful consideration of resource allocation, training of healthcare professionals, and ensuring that the implementation is cost-effective and does not disrupt the overall workflow efficiency [46].

Finally, AI and radiomics should be considered as complementary tools rather than replacements for human expertise in neuro-oncology diagnosis [47]. While these technologies offer valuable insights and assist in decision-making, they should not undermine the role of experienced clinicians [48]. The human aspect of healthcare, such as considering patients’ holistic well-being, ethical considerations, and individual preferences, cannot be solely addressed by AI algorithms and radiomics [49]. In other words, making the proper balance between technology and human expertise is crucial for optimal patient care and outcomes.

## Conclusion

Our study summarized the significant impact of AI in neuro-oncology disease diagnosis and emphasized the potential of radiomics to improve healthcare outcomes, enhance decision-making, and revolutionize patient care. Indeed, the integration of AI and radiomics in diagnosing neuro-oncology diseases presents significant advancements and opportunities in healthcare. While challenges remain, ongoing research and continued collaboration between AI and healthcare experts promise to achieve more improvements in disease diagnosis.

## Conflict of Interest

The authors declare that there are no conflicts of interest.
